# Meiotic behavior of small chromosomes in maize

**DOI:** 10.3389/fpls.2013.00505

**Published:** 2013-12-17

**Authors:** James A. Birchler, Fangpu Han

**Affiliations:** ^1^Division of Biological Sciences, University of MissouriColumbia, MO, USA; ^2^State Key Laboratory of Plant Cell and Chromosome Engineering, Institute of Genetics and Developmental Biology, Chinese Academy of SciencesBeijing, China

**Keywords:** minichromosomes, maize, meiosis, sister chromatid cohesion, synapsis

## Abstract

The typical behavior of chromosomes in meiosis is that homologous pairs synapse, recombine, and then separate at anaphase I. At anaphase II, sister chromatids separate. However, studies of small chromosomes in maize derived from a variety of sources typically have failure of sister chromatid cohesion at anaphase I. This failure occurs whether there is pairing of two copies of a minichromosome or not. These characteristics have implications for managing the transmission of the first generation artificial chromosomes in plants. Procedures to address these issues of minichromosomes are discussed.

Normal chromosomes enter into meiosis following DNA replication. The homologous pairs find each other in the process of synapsis. Recombination exchanges parts of the homologs involving two of the four chromatids present. Then in anaphase I, each member of the pair segregates to opposite poles and into the two cells resulting from meiosis I. In contrast to mitosis, the sister chromatids of each homolog remain attached to each other. At meiosis II the sisters now separate to opposite poles. The consequence of this sequence of events is that segregation of genetic markers on the two members of the pairs of homologs enter into different gametes that will eventually be formed after meiosis. However, small chromosomes as observed in maize do not follow these rules. In this review, we describe the information known about these minichromosomes and what they inform us about meiotic processes. Also, the behavior of small chromosomes impacts how engineered minichromosomes will behave and so information about them is important for practical applications as well.

The first recognition of the unusual behavior of small chromosomes was made by McClintock (1931) in a study of a variety of X-ray induced chromosomal abnormalities. In a little known publication, she described a chromosomal aberration in which the centromere had been removed from a chromosome and was present as a tiny ring chromosome. In the observations about meiosis, she non-chalantly mentioned that the small chromosome separates at meiosis I unlike the other chromosomes. Interestingly, no particular attention was drawn to the fact of this unusual behavior.

In a subsequent paper on the use of ring chromosomes to uncover homozygous deficiencies with a description of their phenotypic effects, McClintock (1938) again notes that the small ring chromosomes show separation at meiosis I instead of at meiosis II. Further, when the same small ring was made to be present twice in the same plant, the two copies, despite being the same, did not exhibit homologous pairing. This observation is the first evidence that small chromosomes do not, or at least seldom, participate in homologous pairing in maize.

Rhoades (1940) discovered a telocentric chromosome composed of the short arm of chromosome 5 that originated from a trisomic 5. It likely was derived via centromere misdivision in the trisomic such that the centromere was divided and the chromosome arm 5S alone remained. During the study of this telocentric chromosome, it was reported that it exhibited sister chromatid separation at meiosis I.

In yet another case, Maguire (1987) examined the small chromosome generated by McClintock (1978) called “tiny fragment.” This chromosome has a centromere of unknown origin together with two excellent kernel markers, *Bronze1* (*Bz1*) and *Shrunken1* (*Sh1*) on the short arm of chromosome 9. This minichromosome did not show pairing with the normal chromosomes 9. At meiosis I, it regularly underwent sister chromatid separation. Thus, in this case, a small linear chromosome failed to exhibit sister chromatid cohesion. Yet another small chromosome involving part of chromosome 10 also showed sister separation at anaphase I (Brock and Pryor, 1996).

## RECENT STUDIES OF MINICHROMOSOMES IN MEIOSIS

**FIGURE 1:**
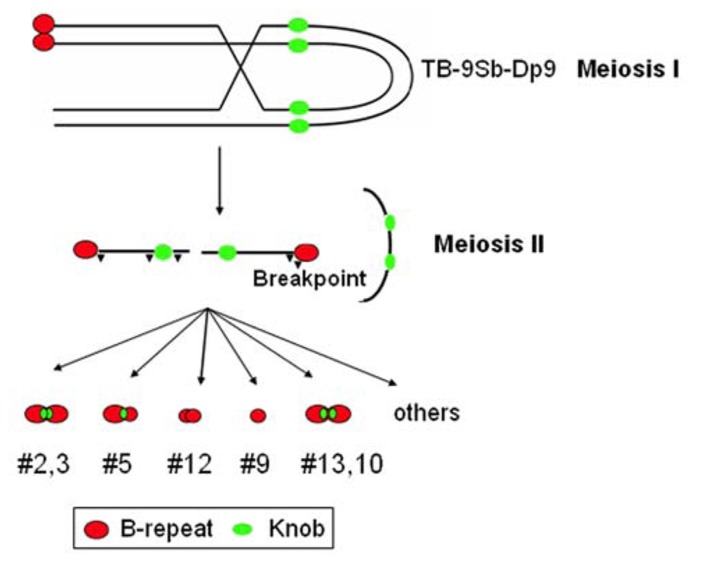
**Initiating the chromosome type BFB cycle to generate minichromosomes.** A translocation between the B chromosome and the short arm of chromosome 9, TB-9Sb, was recombined with a reverse duplication of 9S to generate TB-9Sb-Dp9. This chromosome can recombine with itself as depicted which will join the sister centromeres. At meiosis II the sister centromeres will separate and create a chromatin bridge that will rupture. This broken chromosome will fuse, form a bridge, and break through the gametophyte generation but at the second pollen mitosis the B centromere will undergo non-disjunction. This will place two broken chromosomes in a sperm and thus following the subsequent fertilization event, these two broken chromosomes will set up the chromosome type of BFB cycle that will continue throughout development. Using this approach, Zheng et al. (1999) and Han et al. (2007) produced a collection of minichromosomes described in the text. B repeat is the B chromosome specific sequence present in and around the centromere of the B chromosome. Knob is the repeats of heterochromatin found on maize chromosomes including the short arm of chromosome 9.

A collection of minichromosomes of varying lengths was examined for their behavior in meiosis by Han et al. (2007). This collection consisting of 22 chromosomes was derived from a single progenitor chromosome that was undergoing the chromosome type of the breakage-fusion-bridge (BFB) cycle (**Figure 1**). Zheng et al. (1999) produced the progenitor chromosome which consists of a translocation between the supernumerary B chromosome and the short arm of chromosome 9 onto which had been recombined a foldback chromosome originally recovered by McClintock.

Before describing the behavior of this collection some background is provided. The B chromosome of maize is an extra chromosome that is basically inert (Carlson, 1986). It is not required nor is it detrimental in low copy number. It is preserved in maize populations because it has a drive mechanism. This mechanism consists of two parts. The centromere undergoes non-disjunction at the second pollen mitosis, which produces the two maize sperm. Then the sperm with the B chromosomes preferentially fertilizes the egg as opposed to the polar nuclei in the process of double fertilization. There are at least two factors on the B long arm that are required to be present in order for non-disjunction to occur. One is present at the very tip of the long arm and the other is located within the so-called proximal euchromatin. When either of these factors is missing in the nucleus, then non-disjunction does not occur. These factors act in trans such that a truncated B chromosome that itself cannot undergo non-disjunction can regain that activity in the presence of a full sized normal B. The B chromosome has a specific DNA repeat that in interspersed in and around the centromere, which facilitates identification of the B and any derivatives in cytological preparations (Alfenito and Birchler, 1993).

The BFB cycle was described by McClintock (1939, 1941) following studies of chromosomes that were broken at meiosis. The broken ends can fuse after replication and thus when the sisters separate at the next anaphase, the bridge is broken and the cycle begins again. This “chromatid” type of cycle is active in the gametophyte generation and in the endosperm. If a broken end is present in the sporophyte, then a telomere is added to the broken end and the cycle ceases. In the “chromosome” type of cycle, there are two chromosomes present that are broken and thus they can fuse with each other and therefore can continue to break and rejoin during the sporophytic generation. However, with normal chromosomes the continued chromosomal type of BFB cycle will be so destructive that the plant is killed before it reached maturity.

In order to bypass this complication, Zheng et al. (1999) used the B-A translocation TB-9Sb that, as mentioned, had a foldback duplication of the arm recombined upon it. The goal was to create a situation that would undergo the chromosome type of BFB cycle without killing the plant. The following scenario does that. If in the foldback duplication, recombination occurs, the products of this event will be an acentric fragment and a chromosome in which the sister centromeres are now joined (**Figure 1**). At meiosis I, the B-9Sb-Dp9 chromosome will proceed to one or the other pole. Then at meiosis II, the fragment is released and the sister centromeres proceed to opposite poles generating a chromatin bridge between them, which will break. This broken chromosome will initiate a chromatid type of cycle in the initial gametophyte mitosis but the B centromere will undergo non-disjunction at the second pollen mitosis. Thus, the sperm with the B chromosomes will deliver two broken chromosomes to the zygote. This event establishes the condition for the chromosome type of cycle. The two broken chromosomes replicate and fuse to form a dicentric. Depending on whether the sister chromatids proceed to the same or opposite poles, then either dicentrics will be present in the daughter cells or two broken chromosomes. Because these chromosomes are distinct from the normal chromosome 9 and the other chromosomes, the cycle can continue during development.

When plants that were undergoing this cycle where crossed, the next generation contained a variety of sizes of chromosomes derived from the TB-9Sb-Dp9 progenitor (Zheng et al., 1999; Kato et al., 2005; Han et al., 2007). Some consisted of basically the centromere of the B chromosome but others were of varying lengths. Five were stable dicentrics, which were found to contain one inactive centromere (Han et al., 2006), the first found in plants. This collection of chromosomes was examined for their behavior in meiosis.

**FIGURE 2:**
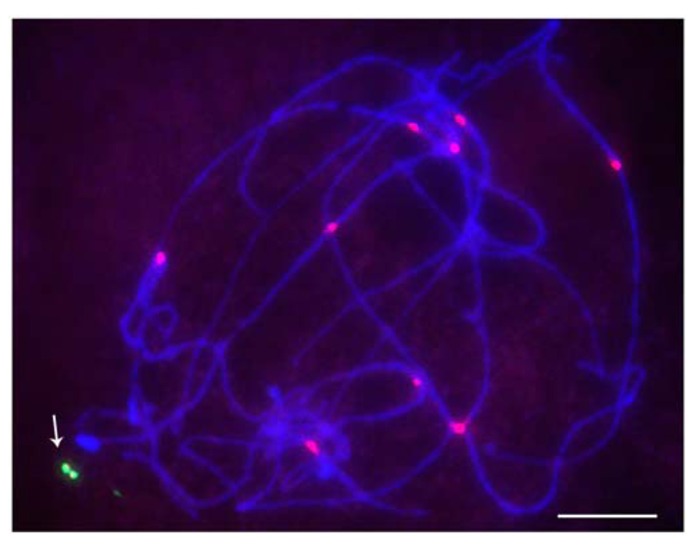
**Pachytene stage with minichromosome revealed by FISH.** Red is CRM, the maize centromeric retrotransposon, and green is the B-specific sequence (ZmBs); arrow indicates the dicentric minichromosome. Bars = 10 μm.

**FIGURE 3:**
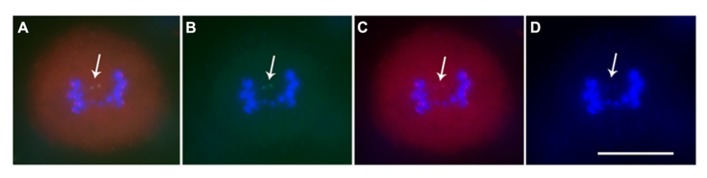
**Immunostaining and FISH for minichromosome. (A)** Merged image; **(B)** Green is ZmBs; **(C)** Red is CENH3, the centromeric histone signals; **(D)** Blue is DAPI. Arrows indicate that the minichromosome sister chromatids separate in meiosis I and each sister has a functional centromere. Bars = 10 μm.

**FIGURE 4:**
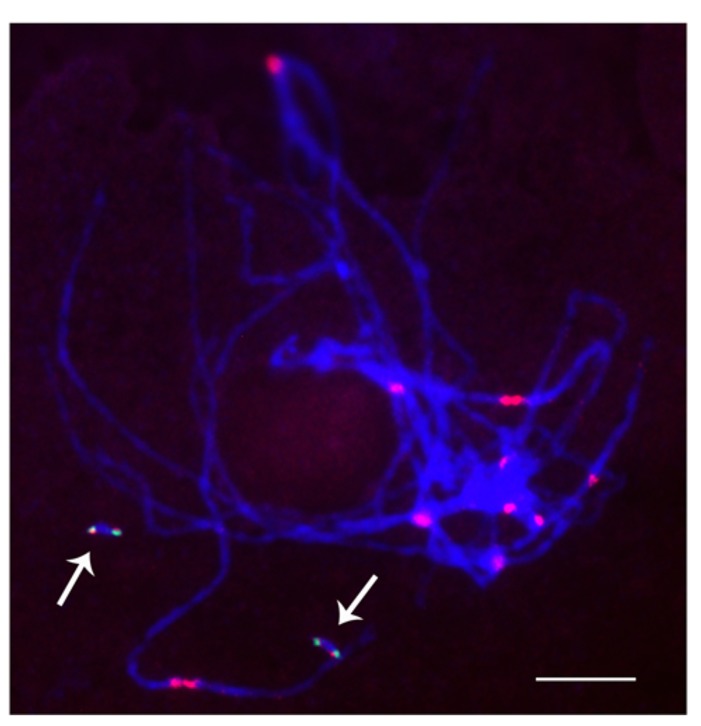
**Cytological analysis of two copies of a minichromosome.** Arrows indicate that the two dicentric minichromosomes do not pair with each other. ZmBs is red and green is knob heterochromatin. Bars = 10 μm.

**FIGURE 5:**
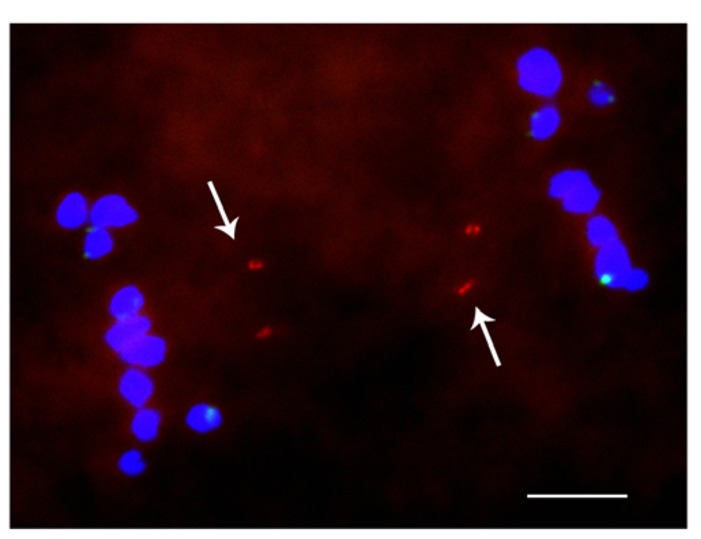
**Cytological analysis of two copies of a minichromosome.** ZmBs is labeled in red; knob heterochromatin is labeled in green. Arrows indicate that the separation of sister chromatids at anaphase I. Bars = 10 μm.

When studied as one copy in meiosis (**Figures 2** and **3**), eight of the 22 derivatives have sister chromatid cohesion at meiosis I as does the normal B chromosome when present as a singleton (Han et al., 2007). In contrast, 14 of the 22 had failure of sister cohesion. When these materials were self-pollinated and individuals with two copies were selected, the pairing of these chromosomes could be examined. The larger versions could find their homologous partner. Those of intermediate size showed pairing in a range of 25–100% of the cells containing two copies. For the tiny minichromosomes, they generally cannot find their pairing partner (**Figures 4** and **5**) although one of the smallest was an exception in that pairing regularly occurred. This small chromosome that showed pairing nevertheless exhibited a failure of sister chromatid cohesion. This fact illustrates that the failure of sister cohesion is not dependent on homologous pairing.

Studies from other species have identified some of the molecules involved with the cohesion properties of chromosomes in meiosis (Kitajima et al., 2004; Vaur et al., 2005; Watanabe, 2005a,b; Kurihara et al., 2006). The Separase enzyme dissolves cohesion at anaphase I allowing the sisters to dissociate. The cohesion complex in mitosis and meiosis is distinct with Rad21, the mitotic component, being replaced by Rec8 in meiosis. The Rec8 homolog in maize has been identified as the *absence of the first division* (*afd1*) mutation (Hamant et al., 2005). In meiosis, the Shugoshin protein protects the centromere so that cohesion is maintained in meiosis I and then dissociates in anaphase II, when Shugoshin is degraded.

Interestingly, immunostaining for Shugoshin of normal B chromosomes and the minichromosomes demonstrated the presence of this protein in both cases during meiosis I even though the small chromosomes had sister separation (Han et al., 2007). Thus, Shugoshin alone is incapable of preventing sister separation. Apparently, chromosome size plays a role, as well as perhaps a need for adjacent pericentromeric regions, for proper establishment of cohesion.

Small chromosomes derived from telomere-mediated chromosomal truncation also exhibit a failure of sister cohesion (Yu et al., 2007; Masonbrink and Birchler, 2012; Gaeta et al., 2013). Minichromosomes derived from both the B chromosome (Yu et al., 2007) or an A chromosome (Gaeta et al., 2013) show the same behavior. A small fragment of chromosome arm 3L with a *de novo* centromere also shows failure of sister cohesion in meiosis I (Fu et al., 2013).

## IMPLICATIONS FOR ENGINEERED MINICHROMOSOMES

The properties of minichromosomes have implications for the use of engineered minichromosomes. The generalized failure of pairing would indicate that a pair of minichromosomes would not segregate from each other at anaphase I but instead would proceed to the poles independently. Secondly, the failure of sister cohesion would also prevent the predictable transmission of a pair of minichromosomes, whether there is pairing or not. Of course, for vegetatively propagated species with a bypass of meiosis, there is no issue because minichromosomes with an endogenous centromere typically have good mitotic stability.

Nevertheless, while these considerations must be taken into account, these obstacles should be able to be overcome. One possible way is to use a truncated B chromosome that still contains substantial portions of the long arm present. These longer B chromosomes do not undergo non-disjunction at the second pollen mitosis because they are missing the distal tip. However, they are large enough to exhibit homolog pairing and to have faithful sister chromatid cohesion at meiosis I (Han et al., 2007). Because they are B chromosomes, they are basically inert and so the chromatin present is unlikely to have any impact on plants. Yet, their termini are engineered in a manner that will allow the addition of new DNA to them in order to grow them as one might prescribe.

A second approach that could overcome this issue would be to place a gametophyte selection on the minichromosome in one copy at every generation. The transmission of a single copy of a minichromosome is usually at a workable frequency. If a gene were placed on the minichromosome that would allow it to survive in the pollen but other grains did not, then a single such engineered minichromosome used as a male parent in each generation would place a full representation of the minichromosome into the next generation. A potential example would be to place the restorer of fertility, *Rf3*, of the cytoplasmic male sterility S (*cms-S*) system onto the minichromosome in a background of *cms-S*. The *cms-S* cytoplasm is a mitochondrial mutation that causes pollen sterility. The *Rf3* restorer acts in individual pollen grains to provide viability. Thus, if a minichromosome carrying *Rf3* were present in a background of *cms-S* and is crossed as a male parent to females carrying the male sterile *cms-S* at each generation, the transmission should be complete at each step.

As the minichromosome manipulations continue to advance, it might become possible in the future to engineer a system to overcome the cohesion and pairing issues. Clearly, in the distant future, if one can contemplate growing back a chromosome to sufficient length to provide cohesion and pairing properties, then such synthetic chromosomes would be expected to transmit well. We do not know, however, at this time how a synthetic chromosome might behave in terms of compaction and other properties in the absence of evolutionary selection. The promise of the engineered chromosome field is that we will learn these parameters in the future.

## Conflict of Interest Statement

The authors declare that the research was conducted in the absence of any commercial or financial relationships that could be construed as a potential conflict of interest.

## AUTHOR CONTRIBUTIONS

James A. Birchler and Fangpu Han wrote the paper.
